# Barriers and facilitators of videoconferencing psychotherapy implementation in veteran mental health care environments: a systematic review

**DOI:** 10.1186/s12913-020-05858-3

**Published:** 2020-11-01

**Authors:** Samuel D. Muir, Kathleen de Boer, Maja Nedeljkovic, Denny Meyer

**Affiliations:** School of Health Sciences, Swinburne University of Technology, Melbourne, Australia

**Keywords:** Telemental health, Veterans, Videoconferencing, Implementation

## Abstract

**Background:**

Whilst treatment for mental health issues has traditionally been conducted in-person, advances in technology has seen a recent growth in the use of online video therapy services to help overcome access-to-care barriers faced by those living in rural locations and those unable to travel. These barriers are particularly apparent in the case of veteran populations, which is the focus of this review. Whilst the research investigating the efficacy of online video therapy to treat mental health issues among veterans is promising, widespread adoption and utilisation of this modality remains low with efforts often failing to progress past the pilot phase to implementation. This review focuses on the implementation of online video therapy in veteran mental health care settings and aims to identify the potential barriers and facilitators relevant to implementing the modality in military organisations.

**Methods:**

A systematic search of three databases (PsycInfo, PubMed, and Web of Science) was conducted. To be eligible for inclusion, studies had to investigate the challenges, lessons learnt, or factors operating as barriers and/or facilitators to the implementation of online video therapy in veteran health care systems.

**Results:**

The initial search revealed a total of 202 articles. This was reduced to 133 when duplicates were removed. After screening the titles and abstracts a further 70 articles were excluded leaving 63 to be retrieved for full review. A total of 10 studies were included in this review. The most commonly reported barriers were related to clinician concerns, logistical problems, and technology. Other barriers included access to resources as well as challenges posed by collaborations, policy and recruitment. Facilitators included experience using the modality and having dedicated staff responsible for promoting and managing the new service (e.g., on-site champions and telehealth technicians).

**Conclusions:**

This review suggests that numerous barriers must be identified and addressed before attempting to implement an online video therapy service in veteran organisations. Further research is needed to establish best practice for implementation, particularly across geographically dispersed sites. It is hoped that the findings of this review will be used to help inform future implementation efforts and research initiatives in this space.

**Supplementary Information:**

**Supplementary information** accompanies this paper at 10.1186/s12913-020-05858-3.

## Background

Whilst treatment for mental health issues has traditionally been conducted in-person in civilian and veteran settings alike, advances in technology has seen a recent growth in the use of telemental health services (e.g., psychoeducational websites, email-based counselling, and online video therapy) [1–3]. Online video therapy (OVT) in particular, has been described as a treatment modality with great potential as it offers a close approximation to the traditional modality of in-person treatment [4, 5]. Videoconferencing technology (e.g., computers, laptops, tablets, smartphones) enables a therapist and client to communicate (visually and audibly) in real-time and engage in therapy despite being in separate geographical locations (i.e., not occupying the same room) via the internet.

The overarching goal of such services is to provide access to quality mental health care to those who need it, particularly for those individuals hindered by access-to-care barriers. Such barriers are particularly pertinent in veteran populations. For example, travelling even short distances to receive in-person treatment can be a challenging task for veterans suffering from posttraumatic stress disorder (PTSD) and/or phobias, who may find the act of travel too distressing (e.g., driving phobias after accidents or roadside bomb attacks) [6]. Research has also found that veterans are reluctant to seek treatment for mental health problems due to stigmatisation fears [7, 8] and potential harm to their careers [9–12]. Hence, it is important that services that overcome these access-to-care issues are explored and, if found effective, implemented.

The evidence-base for OVT in veteran populations continues to grow with results from randomised controlled trials (RCTs) demonstrating promising results, suggesting that veterans receiving treatment via OVT tend to experience a significant decrease in symptomology for a range of mental health problems, including depression [13, 14], anxiety [14] and PTSD [15, 16]. These improvements are not significantly different to those achieved by clients receiving treatment in-person. However, despite a growing body of empirical support, widespread adoption and utilisation remains low with implementation initiatives faced with significant challenges [17–25].

When OVT services are introduced, it is often envisaged as simply a task of introducing new technology [26], however, the logistics of implementing a new modality into existing and complex clinical settings is a difficult task that requires significant planning and change. Moreover, implementation planning is crucial to the effectiveness and sustainability of service deliveries [27, 28]. Despite numerous attempts to introduce and adopt OVT services into existing practices, how to best implement and sustain OVT modalities in veteran mental health care settings is yet to be established.

Veteran mental health care settings present new challenges for OVT due to the strict data security requirements (e.g., no internet access in many offices), increased prevalence of PTSD [29] and the regimented protocols, policies, and procedures often found in these organisations. Organisational resource constraints within veteran mental health care settings have also been identified as critical factors impeding the implementation of OVT in these settings [30]. Moreover, the providers of mental health services in these settings are typically geographically dispersed with each individual centre having their own leaders and stakeholders as well as unique needs and priorities, which can make implementation more challenging [30].

To the authors’ knowledge, there has been no previous attempt to synthesise the literature on OVT implementation in veteran organisations. This review aims to fill this significant knowledge gap in the literature by identifying the potential factors that can hinder and facilitate efforts towards implementing OVT in these settings.

## Methods

This systematic review was reported in accordance with the Preferred Reporting Items for Systematic Reviews and Meta-analysis (PRISMA) statement [31].

### Search strategy

The search was undertaken on May 18, 2018. The search was conducted across three databases: PsycInfo, PubMed, and Web of Science. The search was conducted using the following search terms: (implement*) AND (videoconferenc* OR (video conferenc*) OR (online counselling) OR (online video counselling) OR telehealth OR teleme* OR telepsych*) AND (mental) AND (veteran* OR defence OR military).

### Search criteria

To be eligible for inclusion, studies had to investigate the perspectives and/or experiences of mental health professionals (e.g. clinicians, managerial staff, directors) and/or administrative personnel (e.g., clerical staff, IT staff) in regards to the implementation of OVT. Implementation was defined by the authors as any effort to set up an OVT modality, where an OVT modality represents any service that uses videoconferencing technology (e.g., computer with a webcam) to facilitate treatment for mental health issues (e.g., depression, anxiety, PTSD). Implementation had to occur within a health care service or service provider dedicated to assisting military personnel (e.g., veterans) and/or military personnel families. This particular setting was selected as these organisations (e.g., the US Department of Veterans Affairs; US VA) represent complex geographically dispersed health systems and have been recognised as leaders in the telemental health research space [32].

To be eligible for inclusion, studies had to investigate (or at least report on) challenges, lessons learnt, or factors operating as barriers and/or facilitators to the implementation of OVT, however, this did not need to be the primary focus of the studies. The authors define ‘barriers’ as any factors that hinder the capacity for organisations to implement OVT, while facilitators are defined as any factors that enable the implementation of OVT.

To be eligible for inclusion, studies needed to be published in peer-review journals and be published in English. All research designs were eligible for inclusion.

### Study quality assessment

Study quality was assessed by SM and KdB. Due to the lack of validated quality assessment tools for implementation evaluations an adapted version of the criteria described by Naylor and colleagues [33], originally adapted from Wierenga and colleagues [34], was used (Additional file 1). Items were scored as positive (+ 1), negative (0), or not applicable. When an item was not applicable, that item was excluded from the calculation of that study’s quality rating. A total quality score was calculated for each study by summing the total number of positive items by the total number of applicable items. Scores were then converted to a percentage by multiplying each score by 100. Studies were classified as either strong (> 75%), moderate (50–75%), or weak (< 50%). SM and KdB conducted quality assessments for all reviewed articles. Any disagreements between the reviewers were resolved through discussion.

### Data extraction

Initial search records were recorded in a Word document. Duplicates were removed using the ‘find’ function. Articles were screened at the title and abstract level by SM. Complete reports for the articles that appeared eligible for inclusion were retrieved and stored in a Mendeley folder. When an abstract contained insufficient information to determine whether it satisfied the inclusion criteria, the full paper was retrieved and read.

The data extraction was performed by SM using a Google form. The form was developed by the authors and was used to extract the necessary information from each of the studies identified for inclusion in the review. The form collected information on author(s), year of publication, study design, setting, population, results (i.e., barriers and facilitators), and discussion (e.g., lessons learnt and recommendations).

### Data synthesis

To analyse the included studies, an inductive thematic analysis was conducted. The analysis followed the method described by Braun and Clarke [35] and involved six steps: 1) data familiarisation; 2) initial code generation; 3) theme searching; 4) theme revision; 5) theme definition and naming; and 6) reporting. Specifically, after familiarising (step 1) and coding the data extracted from the included studies (step 2), factors were categorised as either barriers or facilitators (step 3). Factors were then counted by frequency before being categorised further into sub-groups of barriers and facilitators (step 4) and named (step 5) (see Tables 1 and 2).

**Table 1:** Themes of factors operating as barriers

Theme	Description
Clinical staff	
Scepticism/attitudes	Clinicians held a negative attitude towards the OVT modality [17, 19, 22, 23, 25]
Lack of training/experience	Clinicians lack training and experience using OVT [19, 22]
Lack of need for OVT	Clinicians were unaware of the need for OVT [22]
Lack of time	Clinicians did not have time to familiarise themselves with the OVT modality [22]
Logistical barriers	
Scheduling	Services experienced difficulties incorporating OVT sessions into existing schedules [18, 19, 22]
Staffing	Services did not have enough staff to dedicate to OVT implementation [17–19, 22]
Logistical support	Services lacked the logistical support required to implement OVT [22]
Technology	
Connection	Services lacked the necessary internet infrastructure to implement OVT [19, 22, 24, 36, 37]
Set up	Services lacked the expertise to install the OVT software and hardware [17, 19, 22]
Technical support	Services lacked the necessary technical support to oversee implementation [17, 22]
Resources	
Space	Services lacked the required space to set up dedicated OVT rooms [18, 24]
Equipment	Services lacked the required equipment (e.g., headphones and webcams) [18, 24]
Funding	Services lacked the funding required to implement and sustain the OVT service [19]
Collaboration	
Bureaucratic delays	OVT implementation was delayed due to administrative and ethical issues between collaborators [19, 25]
Communication	OVT implementation was delayed due to communication issues between collaborators [19]
Policy	
Modifying existing policy	Services needed to modify existing policy to implement OVT [19]
Clients	
Recruitment	Services and researchers struggled to recruit clients to participate in OVT trials [17, 19, 22]

**Table 2:** Themes of factors operating as facilitators

Theme	Description
Clinical staff	
Dedicated OVT clinicians	Services with a select group of clinicians dedicated to engaging in OVT [19, 22]
Experience with OVT	Clinicians with prior experience using OVT [17, 19, 22]
Motivation	Clinicians that were motivated to engage in OVT [17]
Training	Clinicians that had received OVT training prior to implementation [22, 23, 37]
Other staff	
OVT champions	Services with dedicated staff championing the OVT modality [18, 19, 22, 23, 25, 38]
Telehealth coordinators	Services with dedicated telehealth coordinators responsible for overseeing implementation of the modality [23, 38]
Telehealth technician	Services with dedicated telehealth IT specialists to oversee the implementation of software and hardware [19, 36]
Stable personnel	Services with low attrition of staff [19, 23, 37]
Leadership support	Services with leadership that prioritise the need of OVT and facilitate implementation [23, 38]
External facilitators	Services that employ external groups to oversee OVT implementation [23]
Technology	
Ease of use	OVT software that is easy to use [18]
Implementation strategy	
Tailored approach	Strategies that are tailored to each individual service provider [23, 38]
Pilot site	Services that take a stepped approach to implementation, commencing at a single pilot site [25]
Standardised procedures	Services that standardise implementation procedures [17]

## Results

### Review statistics

**Fig. 1:**
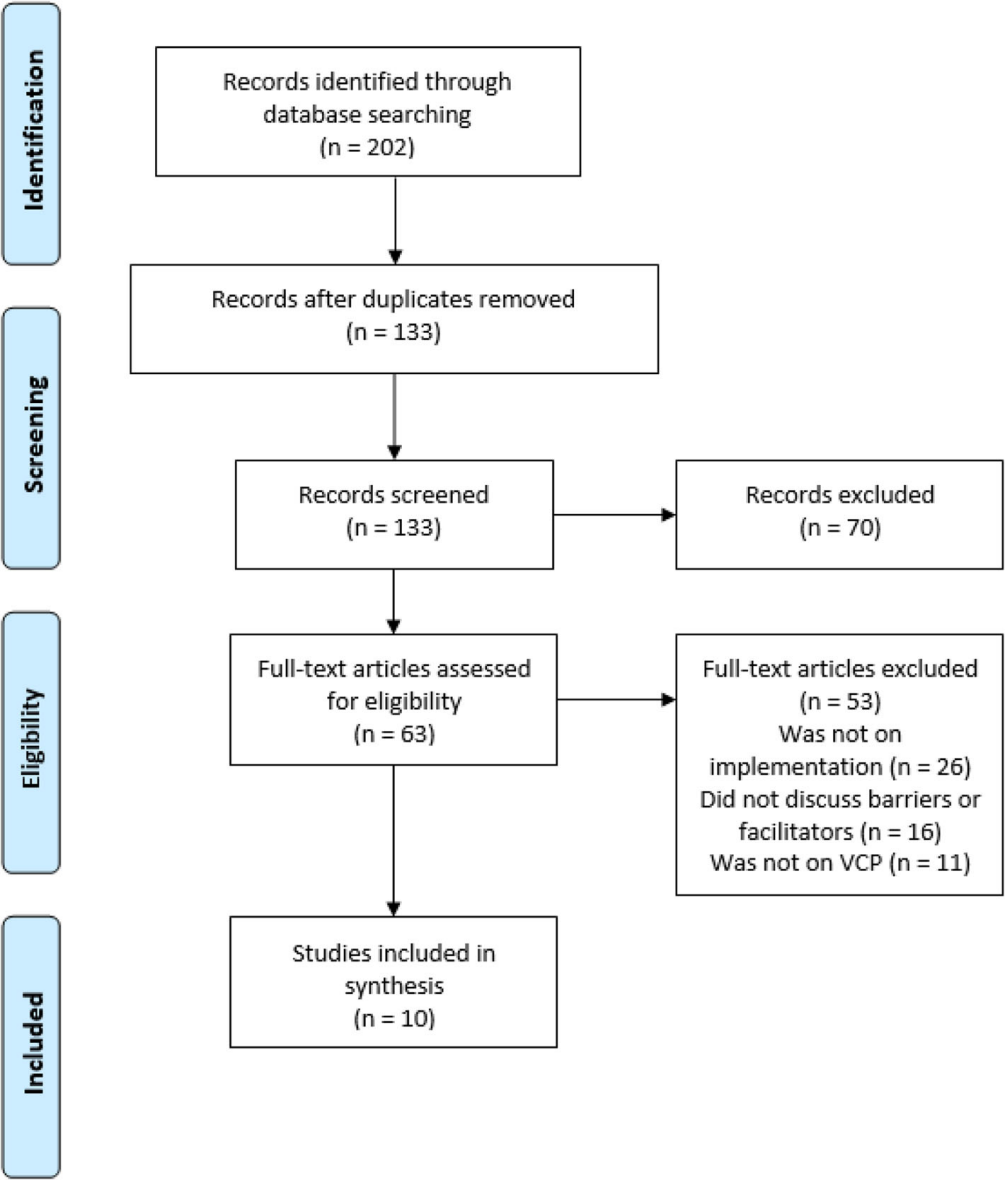
PRISMA flowchart

The initial search revealed a total of 202 articles. This was reduced to 133 when duplicates were removed. After screening the titles and abstracts a further 70 articles were excluded leaving 63 to be retrieved for full review. A PRISMA flowchart [31] was created to illustrate the number of articles found at each stage of the data acquisition and the number of articles that were excluded at each stage (see Fig. 1). No additional papers were retrieved after reviewing the reference lists of the included studies. Ten studies were included in the thematic analysis (see Additional file 2).

### Study characteristics

All studies included in this review were published in 2005 or later and investigated OVT in the US VA. The majority of studies presented findings from semi-structured interviews [18, 19, 22, 24, 36], or were case study reports [23, 25, 37]. One study [17] collected data using a survey and there was also one review [38] included. Sample sizes ranged from 12 to 40 and consisted of either clinical staff or a mixture of clinical staff and other personnel included in the implementation of OVT (e.g., IT and administrative staff, organisational leaders and other stakeholders).

### Study quality assessment

Study quality was weak overall; this was likely as a consequence of studies using only post-implementation data collection methods. One study [18] received a strong rating, three studies [19, 22, 24] received moderate quality assessment scores while the remaining five studies received weak scores [17, 23, 25, 36, 37]. One study [38] was a general review of the literature and was unable to be assessed.

### Barriers and facilitators

Factors were categorised as either barriers or facilitators of OVT. Seven factors were identified as barriers (see Table 1) while four facilitating factors were found (see Table 2). The most common barriers reported were clinician-related, and specifically, clinician attitudes. Interestingly, clinical staff were also reported to be one of the most common facilitators of OVT implementation (see Table 2). Other facilitators included staff other than clinicians (e.g., on-site OVT champions), technology, and implementation strategies.

### Thematic analysis

#### Clinical staff

Clinicians who have not experienced OVT appear to have more concerns about the effectiveness and need for the modality. Such clinicians reported concerns regarding the effectiveness of the modality, impact on the therapeutic relationship, their familiarity with the technology, dealing with high-risk clients, and concerns with data security issues [17, 19, 22, 23]. However, studies in this review also found clinician-related factors to be facilitators of OVT implementation. It appears that once clinicians have been exposed to OVT, their concerns or scepticism towards the modality tend to dissipate [17, 19, 22, 23]. This point is best illustrated by the findings from Interian and colleagues [22] who conducted qualitative interviews with US VA clinicians and staff (*n* = 33) involved with the implementation of a OVT modality to help veterans with bipolar disorder. They found that perceived barriers and facilitators of OVT implementation varied depending on staff members’ level of experience with OVT. Those with no OVT experience expressed the greatest concerns about the fit/compatibility between OVT and current practice, while those who had used OVT previously reported the least amount of concern. Similar patterns were reported across several of the other included articles with studies reporting that for those staff who do hold initial concerns about the to-be-implemented OVT modality, once they are exposed to the technology, their attitudes and satisfaction levels tend to increase and concerns quickly fade [17, 19, 23].

#### Logistical barriers

Logistical tasks (e.g., inadequate staffing, scheduling, overwhelming workloads, staff changes) were often identified as barriers to OVT implementation [17, 20]. Adler and colleagues [17] reported that all six sites involved in a pilot study of OVT suffered delays to implementation due to administrative barriers. Logistic support was also found to be a common facilitator of OVT implementation [19, 23, 36, 37]. Studies reported that employing staff with roles dedicated to the OVT modality (e.g., telehealth coordinators and technicians) helped facilitate implementation. These roles typically involved providing access to and support from leadership as well as giving guidance with setting up and navigating the OVT technology and access to ongoing technical support.

#### Technological obstacles

Several studies reported technological-related issues to be a barrier of OVT implementation, the most common of which were connectivity issues [19, 22, 24, 36, 37]. Studies also reported issues regarding the set-up of the equipment required to engage in videoconferencing [17, 19, 22] as well as a lack of technical support [17, 22]. Other studies were seemingly able to overcome these issues by employing staff dedicated to overseeing the online modality [19, 23, 36, 38].

#### Resources

A common challenge noted in previous studies was running into unanticipated organisational constraints, such as procuring and maintaining resources as well as retaining expert personnel who possess the knowledge required to implement OVT [17–19, 22, 24]. Adler and colleagues [17] described how implementation in their project was delayed by encountering constraints including limited space, lost work orders to install equipment, misplaced equipment, and difficulties setting up and supporting new services. Similarly, Moreau and colleagues [24] described numerous challenges that stakeholders experienced with implementing OVT services, including access to OVT equipment (e.g., computers, monitors, and webcams) and appropriate space (e.g., dedicated rooms to conduct OVT sessions).

#### Multi-organisational challenges

Communication problems and delays caused by bureaucratic challenges were cited as barriers in multi-organisational OVT collaborations [19, 25]. Despite these challenges, personnel involved in these studies tend to recognise that collaborations are necessary to implement new services and conduct related research successfully [19, 25].

#### Facilitators

Despite the significant challenges of implementing OVT outlined above, several facilitators have also been identified. For example, in their multi-site implementation effort, Lindsay and colleagues [23] were able to identify some distinguishing features between sites where facilitation was high (i.e., good) and low (i.e., poor). High-facilitation sites were those that engaged in the implementation process early, committed appropriate resources to OVT services (e.g., dedicated program and IT staff) and tended to have a clinician dedicated solely to providing OVT. Similarly, in their implementation study, Interian and colleagues [22] found that sites that witnessed successful implementation had the following characteristics:

- A team of providers dedicated solely to providing mental health treatment via the new OVT modality;
- Site members actively engaging clients by promoting the OVT service at local community events;
- Regular emails to clinical staff promoting the study and asking for expressions of interest to receive training and participate in the study;
- Employing local “champions” within the site to promote the study and provide ongoing logistical support.

Sites with the greatest difficulty implementing OVT were those who perceived themselves to have less access to support and less recognition of a need for implementation [22, 23].

#### On-site champions

On-site champions were described in several studies as being crucial for successful implementation [17–19, 22, 23, 38]. Studies described the role of the “champion” to typically involve promoting awareness and the benefits of the modality. Champions were assigned a variety of tasks, from making presentations at local clinic meetings to encouraging visibility for projects and answering questions posed from clinical staff. Beyond encouragement and promotion, the champions were also assigned more hands-on roles, for example, demonstrating how to set up and use the OVT software, distributing resources, and providing a gateway between clinical staff and implementation teams and external research teams. Studies reported that employing on-site champions could increase clinician motivation, willingness to try and eventual adoption of the modality [17, 19, 22, 23].

#### External facilitators

External facilitators were also reported to be beneficial with implementation [23]. An external facilitator is typically an individual (or team) specifically trained in implementation that is responsible for helping sites solve problems and liaising with various stakeholders to ensure that all implementation needs are addressed (e.g., accommodating newly hired staff, acquiring new equipment, etc.). Lindsay and colleagues [23] surveyed clinicians involved in their study with results suggesting clinicians found the external facilitation model to be very helpful in implementing the OVT service.

#### Implementation strategy

Researchers noted that taking a stepped approach was a crucial strategy for ensuring successful implementation [23, 38]. Conducting initial pilot and feasibility studies was also recommended [25, 38]. Shore and Manson [25] described a framework they used to implement OVT. Their model consisted of six steps (needs identification, infrastructure survey, partnership, structure configuration, pilot, and solidification). The researchers highlighted that the timeline for implementation at each site was largely dependent on the participation and buy-in from staff within those sites. However, in the case of all sites they were ultimately successful in implementing and sustaining OVT [25].

## Discussion

### Summary of findings and recommendations

This systematic review focused on the implementation of OVT in veteran mental health care settings. Specifically, the review has identified and synthesised the results from ten studies investigating OVT implementation, all of which were conducted by the US VA. The most commonly reported barriers were related to clinician concerns, logistical obstacles, and technology. Other barriers included access to resources as well as challenges posed by multi-organisational collaborations, policy and recruitment. In this discussion, particular attention is paid to the impact of factors that are more specific to veteran mental health services, such as the prevalence of PTSD amongst clients, the importance of data security, resource constraints and the geographical dispersion of service providers.

### Clinical staff

For clinicians to adopt the OVT modality, it appears that motivation, training and communication are critical. It is not surprising that clinicians tend to hold concerns about OVT as going from seeing a client in-person to engaging with them via an online videoconferencing system is a significant change for clinicians, many of whom may not have received training specific to the online modality. This concern may be heightened in veteran mental health settings considering the increased prevalence of PTSD [29] and the additional unique potential risks posed by OVT delivery [39]. This concern is not restricted to clinicians in veteran settings and has been frequently cited as a barrier in the wider OVT literature [40–42]. However, this review found that once clinicians began engaging with the modality, concerns tend to dissipate. Once again, this is consistent with the wider OVT literature which has shown that even those clinicians who are initially hesitant to use the technology are able to adapt successfully and embrace OVT once it is introduced to them [43–45]. Organisations looking to implement OVT may want to consider involving only those clinicians who are already interested or experienced in providing OVT as a first step, and then invite those clinicians with limited experience to start engaging only after the modality has been implemented and they have witnessed some positive outcomes.

Studies also reported a lack of training in OVT to be a barrier for OVT implementation in veteran mental health services [20, 22]. This is consistent with previous reviews of US VA initiatives that found a lack of training to be a barrier in the implementation of system-wide evidence-based treatments [30, 45]. A lack of training may result in a lack of confidence in engaging in the modality, which may ultimately lead to low uptake. Consequently, it is no surprise that experts stress the importance of providing OVT training and guidelines to clinicians (and clients) prior to treatment [26, 46–48]. Several different approaches were described in previous studies for training clinical staff to engage in OVT ranging from training manuals and half-day in-person workshops to online programs [17, 37]. All clinicians participating in OVT should be trained on how to use the technology before learning how to engage in OVT treatment and this training must be completed before a clinician can engage in OVT with clients. For studies involving implementation at more than one site, it is important that training be standardised across sites [17, 37].

Clinicians should have access to resources and peer support to assist with any issues that arise post-training and throughout their time delivering OVT. In particular, training for OVT with veterans experiencing PTSD is a priority as practices unique to PTSD treatment (e.g., exposure therapy) may be perceived to be more difficult and involve more risk than standard practice. Further research is needed in order to understand the specific concerns that clinicians before training programmes can be developed and optimized for engaging in OVT therapy.

### Logistical concerns

OVT implementation typically involves substantial coordination across systems and departments (e.g., clinical, clerical and IT) and the demands are often underestimated in terms of time and complexity [26]. It was not surprising then that logistical tasks (e.g., inadequate staffing, scheduling, overwhelming workloads, and staff changes) were often identified as barriers to OVT implementation. To implement OVT effectively, organisations should ensure that tested administrative infrastructure and protocols are in place and that additional resources are available should further support be required. This is particularly important because of the geographically dispersed nature of veteran mental health services, making the duplication of resources and co-ordination of logistical processes a special challenge.

### External facilitators

As demonstrated in this review, the process of implementing an OVT service into existing clinical practice is a challenging task that is often underestimated in terms of its complexity. Government organisations are often working at capacity with staff often reporting little time to take on additional tasks required for implementation on top of their day-to-day roles [49–51]. Such resource constraints (e.g., staffing and logistical support) make the use of external facilitators particularly important to consider. Facilitation is a strategy that allows interventions to be tailored to enable change or make adopting a new practice easier. This has been used with great success in recent times in the US VA [23, 52–54]. For example, Crowley and colleagues [52] conducted a pilot study to evaluate the feasibility and effectiveness of using existing VA clinical staffing and equipment to deliver a home-based telemedicine intervention for veterans with diabetes. In their study, the intervention was implemented by members of the US VA (internal facilitators) while the research staff (external facilitators) managed the research tasks (e.g., randomisation, outcome analysis) [52]. Dividing the implementation tasks and dedicating specific staff to the implementation can help ease the burden on staff and ensure successful implementation [53].

### Implementation strategy

Researchers have advised the adoption of a stepped implementation process, beginning with a small-scale, manageable pilot study [23, 25, 53, 55]. The lessons learned from a single pilot location can then be used to help facilitate implementation at additional sites. This would allow the implementation team to identify any barriers and come up with modifications to procedures before attempting to implement the service more broadly.

### On-site champions

As described earlier, using on-site champions has been identified as a vital part of making implementation successful [17, 18, 23, 26, 38, 45, 53]. Their primary role is to promote awareness of the initiative and to encourage the adoption of the modality. Champions play a crucial role assisting with the process to ensure OVT integration is as smooth as possible. Champions should provide leadership and a gateway for communication between clinical staff and the implementation team. Previous research has demonstrated that having an expert on-site providing guidance, peer supervision and consultation appears to be one of the most effective ways to ensure that staff adopt the modality [17, 56, 57]. Having on-site champions also alleviates the need for external agents to travel site-to-site to assist with implementation. Hence, the geographically dispersed nature of veteran mental health services make on-site champions of OVT particularly important.

### Fostering buy-in

A common theme extracted from the reviewed studies centred upon the notion of buy-in. The implementation of a new modality will not be successful if those responsible for engaging in the intervention do not support its introduction [58]. Nor will implementation be successful if the new intervention does not fit within the existing ideological framework of the organisation. Clinicians are critical to the utilisation of OVT and often serve as the initial gatekeepers to its implementation, hence, buy-in from these personnel is crucial. However, the introduction of new technology will have a significant impact on not only clinicians, but also clerical and IT staff. A motivated, supportive and adaptable cultural environment is therefore critical.

A recent review of US VA evidence-based PTSD treatment implementation efforts identified cultural fit to be an important facilitator [45]. However, creating a culture that is receptive to change is easier said than done. Research has found that frequent communication from leadership in regards to informing all staff of the need for OVT and providing regular updates on implementation has been found to be critical to implementation success and the fostering of buy-in [23, 25, 38, 53].

Hence, as a first step, organisations seeking to implement OVT should prioritise a transparent approach with staff and provide as much support as possible throughout the implementation process. In line with our prior recommendation regarding clinician involvement, organisations should ensure that participation in OVT delivery is voluntary and that only those clinicians who desire to engage in the new modality are included in the implementation. This will help improve the likelihood of a successful implementation, in that the clinicians will have a vested interest in making things work. In addition, such a strategy should also help reduce the resource constraints that have been found to plague implementation efforts in this space [30] in that not all clinicians will require additional resources in the form of software and additional time for learning how to engage in OVT and the associated technology.

#### Limitations

This systematic review identified and reviewed OVT implementation publications from veteran organisations only and was able to identify studies only from the US VA. There are several possible explanations for this. For example, the databases searched and the narrow search terms used may have resulted in our review being unable to identify OVT implementation papers from other countries. Alternatively, it may be that the USA is the only country publishing implementation papers relevant to the scope of this review (i.e., OVT for veterans). This is not to say that service providers in other countries are not implementing OVT for veterans, but they may not be publishing research on implementation. Despite the US VA being a complex and geographically dispersed organisation that is a leader in telemental health, the findings of this review may not be able to be generalised outside of this organisation to other veteran organisations or civilian settings. It is recommended that researchers consider consulting scholars and practitioners from veteran mental health services based in other countries to yield insights and additional research publications to increase the scope of future reviews for this population. However, the findings from this review may assist other organisations who share some common features with US VA and are considering implementing an OVT service. In addition, despite searching three databases, it is possible that further applicable publications were missed. The lack of search terms for specific mental health issues (e.g., “depression”, “anxiety”) may have limited the identification of additional studies present in the databases that were searched. This review was also limited to peer-reviewed articles published in English.

#### Further study

Although the evidence base for OVT efficacy is growing, best practice for how to implement this modality is yet to be established for veteran mental health services. Researchers implementing and investigating the efficacy of OVT interventions should look to not only evaluate mental health outcomes of clients, but also include plans to evaluate the effectiveness of implementation strategies used. To maximise potential for successful implementation of OVTs it is recommended that researchers and practitioners adopt the Videoconferencing Psychotherapy Implementation model [59] which addresses the barriers and facilitators identified in this review. Evaluation studies will assist researchers and mental health professionals to optimise future implementation efforts and enhance sustainability of the modality.

This systematic review adds to the existing implementation evidence base populated by other similar reviews on evidence-based intervention implementation [60] and telemedicine implementation [21, 61]. To the authors knowledge, this is the first systematic review published investigating the barriers and facilitators specific to OVT implementation. However, the review was restricted to OVT implementation in veteran mental health care settings. Therefore, further research is needed to review OVT implementation efforts in other health care settings (e.g., civilian and independent health care organisations) and investigate whether these are comparable with the factors identified in this review.

## Conclusion

A major challenge to the implementation of OVT in veteran organisations is identifying and overcoming the barriers associated with integrating the service into existing practices. This review identified several obstacles that need to be overcome for efforts involving the implementation of OVT to be successful in veteran organisations. Despite their presence, these challenges have not prevented continued growth in the OVT space with studies demonstrating that, with proper training, resourcing, staffing, and buy-in, OVT services can be successfully implemented in these settings [19, 22, 23]. However, such challenges should not be ignored and should not be underestimated in terms of their complexity and time required to action. It is hoped that the findings of this review will be used to help inform future OVT implementation efforts and research initiatives. However, further research is needed to establish best practice for OVT implementation in veteran organisations.

## Supplementary Information

**Additional file 1.** Study quality assessment measure.

**Additional file 2.** Descriptions of included studies.

## Data Availability

All data generated or analysed during this study are included in this published article and its supplementary information files.

## Abbreviations

IT: Information technology
OVT: Online video therapy
PRISMA: Preferred Reporting Items for Systematic Reviews and Meta-analysis
PTSD: Posttraumatic stress disorder
RCT: Randomised controlled trial
US: United States
VA: Veterans Affairs
