# Supplementary material for: Barriers and facilitators of videoconferencing psychotherapy implementation in veteran mental health care environments: a systematic review
Source: BMC Health Serv Res. 2020 Nov 1;20:999. doi: 10.1186/s12913-020-05858-3 (PMC7603749; doi:10.1186/s12913-020-05858-3)
Supplement: Supplementary file 1 — Additional file 1. Study quality assessment measure. [file 12913_2020_5858_MOESM1_ESM.docx]

**Additional file 1. Process measures quality assessment***

| **Item** | **Evaluation Criteria** |
| --- | --- |
| 1) Level of evaluation | Positive if implementation was evaluated on two or more levels (e.g., client, clinician, clerical staff, leadership) |
| 2) Definition of process variables | Positive if process variables were adequately described |
| 3) Process variables | Positive if four or more process variables were reported |
| 4) Data collection | Positive if two or more techniques were used (e.g., phone interview, survey) |
| 5) Timing of data collection | Positive if process variables were measured on multiple occasions (e.g., pre, during and/or post implementation) |
| 6) Quantitative process variables | Positive if quantitative process outcomes were assessed using methods of acceptable quality |
| 7) Qualitative process variables | Positive if qualitative study design was adequately described (e.g., participant selection, setting, data collection) |
| 8) Outcome related to implementation | Positive if outcomes were evaluated in the context of implementation quality |

*Process measures quality assessment adapted from Wierenga et al.^34^
