# Supplementary material for: Barriers and facilitators of videoconferencing psychotherapy implementation in veteran mental health care environments: a systematic review
Source: BMC Health Serv Res. 2020 Nov 1;20:999. doi: 10.1186/s12913-020-05858-3 (PMC7603749; doi:10.1186/s12913-020-05858-3)
Supplement: Supplementary file 2 — Additional file 2. Descriptions of included studies. [file 12913_2020_5858_MOESM2_ESM.docx]

**Additional file 2** Descriptions of included studies

| Author & year | Title | Organisation | Problem & population | Findings (barriers and facilitators) |
| --- | --- | --- | --- | --- |
| Adler et al.^17^ | A pilot project to improve access to telepsychotherapy at rural clinics | US VA | VCP for US veterans (no specific illness) | Barriers: organisational constraints and administrative barriers  Facilitators: establishing organisational practices and clinician motivation and attitudes |
| Azevedo et al.^36^ | Piloting specialized mental health care for rural women veterans using STAIR delivered via telehealth: Implications for reducing health disparities | US VA | STAIR (Skills Training in Affective and Interpersonal Regulation); a 10-week skills building program designed to help veterans take an active role in managing their well-being. Specifically aiming to reduce PTSD-related symptoms and increase social engagement | Barriers: technological difficulties  Facilitators: telemental health technicians |
| Bauer et al. ^18^ | Implementing and sustaining team-based telecare for bipolar disorder: Lessons learned from a model-guided, mixed methods analysis | US VA | VCP for US veterans with known or suspected bipolar disorder | Barriers: space, equipment, staffing and scheduling  Facilitators: consult content, ease of use, and national infrastructure. |
| Brooks et al.^19^ | The diffusion of telehealth in rural American Indian communities: A retrospective survey of key stakeholders | US VA | VCP for American Indian veterans (no specific illness) | Barriers: clinician scepticism  Facilitators: clinician experience using VCP, VCP champions |
| Interian et al.^22^ | Evaluating the implementation of home-based videoconferencing for providing mental health services | US VA | VCP for US veterans (no specific illness) | Barriers: Lack of experience with VCP and logistical support  Facilitators: Previous experience with VCP, VCP champions |
| Lindsay et al.^23^ | Implementation of video telehealth to improve access to evidence-based psychotherapy for posttraumatic stress disorder | US VA | VCP for US veterans with PTSD | Barriers: clinician scepticism  Facilitators: external facilitation, VCP champions |
| Moreau et al.^24^ | The use of telemental health to meet the mental health needs of women using Department of Veterans Affairs services | US VA | VCP for US women veterans (no specific illness) | Barriers: connectivity, space, equipment  Facilitators: staff enthusiasm |
| Palyo et al.^37^ | Tele-pain management: Use of videoconferencing technology in the delivery of an integrated cognitive–behavioural and physical therapy group intervention | US VA | Group-VCP for US veterans with chronic pain and co-occurring mood disorders | Barriers: connectivity  Facilitators: training and stable personnel |
| Shore & Manson^25^ | A developmental model for rural telepsychiatry | US VA | VCP for American Indian veterans (no specific illness) | Barriers: clinician scepticism and bureaucratic-related delays  Facilitators: VCP champions and pilot studies |
| Shore et al.^38^ | Review of American Indian veteran telemental health | US VA | VCP for American Indian veterans (no specific illness) | Facilitators: VCP champions and stepped implementation |
